# Endoscopic or combined management of post-surgical biliary leaks: a two-center recent experience

**DOI:** 10.1007/s00464-024-11243-6

**Published:** 2024-10-09

**Authors:** Dario Quintini, Giacomo Emanuele Maria Rizzo, Ilaria Tarantino, Giacomo Sarzo, Alberto Fantin, Roberto Miraglia, Luigi Maruzzelli, Dario Ligresti, Lucio Carrozza, Gabriele Rancatore, Salvatore Gruttadauria, Umberto Cillo, Francesco Ferrara, Mario Traina

**Affiliations:** 1 Endoscopy Service, Department of Diagnostic and Therapeutic Services, IRCCS - ISMETT, Palermo, Italy; 2 Department of Surgical, Oncological and Gastroenterological Sciences, University of Padua, Padua, Italy; 3 Gastroenterology Unit, Veneto Institute of Oncology IOV-IRCCS, Padua, Italy; 4 Department of Precision Medicine in Medical, Surgical and Critical Care (Me.Pre.C.C.), University of Palermo, Palermo, Italy; 5 OSA General Surgery, Padua University Hospital, Padua, Italy; 6 Radiology Services, IRCCS-ISMETT, Palermo, Italy; 7 Department for the Treatment and Study of Abdominal Diseases and Abdominal Transplantation, IRCCS-ISMETT, UPMC (University of Pittsburgh Medical Center), Palermo, Italy

**Keywords:** Biliary leaks, Biliary fistula, Post-surgical leak, Post-operative leak, ERCP, Rendez Vous

## Abstract

**Background and Aims:**

Post-surgical biliary leaks (PSBL) are one of the most prevalent and significant adverse events emerging after liver or biliary tract surgeries. Endoscopic retrograde cholangiopancreatography (ERCP) alone or combined with another approach (Rendez Vous) as treatment of PSBL obtains optimal outcomes due to the possibility of modifying the resistances in the biliary tree.

**Methods:**

A retrospective double-center study was conducted in two tertiary centers. Consecutive patients who underwent at least one attempt of PSBL correction by ERCP or Rendez Vous procedure between January 2018 and August 2023 were included. The primary outcome was overall endoscopic clinical success. In contrast, the secondary outcomes were hospital stay exceeding five days and endoscopic clinical success with the first endoscopic procedure at the tertiary center. Both univariate and multivariate analyses were used to assess outcomes.

**Results:**

65 patients were included. Patients with one or multiple) leaks had more possibility to achieve the endoscopic clinical success compared to those affected by the association of leaks and stricture (96% vs 67%, *p* value 0.005). Leaks occurring in the main biliary duct had less probability (67%) to achieve the overall endoscopic clinical success compared to those in the end-to-end anastomosis (90%), in the resection plane or biliary stump (96%) or first or secondary order biliary branches (100%, *p* value 0.038). A leak-bridging stent positioning had more probability of achieving the endoscopic clinical success than a not leak-bridging stent (91% vs 53%, *p* value 0.005).

**Conclusions:**

ERCP and Rendez Vous procedures are safe and effective for treating PSBL, regardless of the type of preceding surgery, even if technical or clinical success was not achieved on the first attempt. A stent should be placed, if feasible, leak-bridging to enhance treatment efficacy.

**Graphical abstract:**

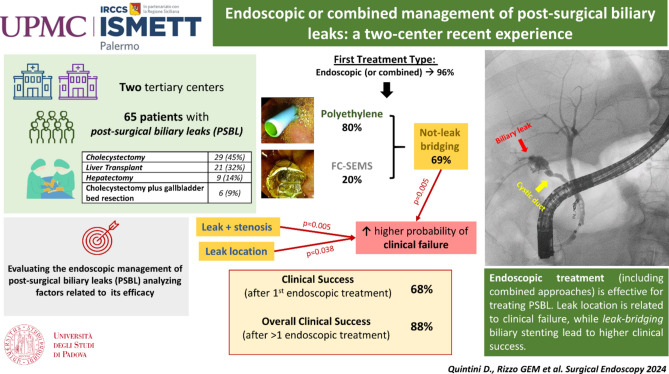

**Supplementary Information:**

The online version contains supplementary material available at 10.1007/s00464-024-11243-6.

Liver and biliary tract surgeries are pivotal in managing a broad spectrum of benign and malignant hepatic and biliary disorders. A well-known and serious postoperative complication is the occurrence of biliary leaks. In recent decades, notable progress has been made in surgical techniques and postoperative care [1, 2]. Nonetheless, biliary leaks continue to be a persistent challenge, contributing to significant morbidity and mortality rates. These leaks are linked to multiple risk factors [3–5] such as the type of surgery performed: post-cholecystectomy leaks have an incidence rate of approximately 0.4–1.5% [6], post-hepatectomy leaks range from about 3–20% [1], or 2–43% if combined with biliary reconstruction [4], while post-orthotopic liver transplantation (OLTx) leaks occur in about 2–25% of cases [7]. Currently, there is no universally standardized algorithm for managing biliary leaks. Endoscopic Retrograde Cholangiopancreatography (ERCP) has increasingly become the preferred initial intervention for most biliary leaks [8, 9]. Its appeal lies not only in its minimally invasive nature but also in its dual diagnostic and therapeutic capabilities. The treatment aims to reduce the ampullary resistances, thereby facilitating the transpapillary flow of bile and minimizing leakage. Stenting, particularly, has proven to be exceptionally effective. The choice between sphincterotomy alone, plastic stents, and the more frequently used fully covered self-expandable metal stents (FCSEMS) is not standardized and typically depends on the leak’s location and characteristics [8–10]. Sphincterotomy alone could be considered in the presence of a minor leak in association with choledocholithiasis. In young patients with a minor leak, stenting alone can be a good choice to preserve the biliary sphincter. Or even, for post-cholecystectomy leaks, Ahmad et al. [11] propose the use of FC-SEMS or multiple plastic stents in post-cholecystectomy leaks if a previous attempt with a 10Fr plastic, with or without sphincterotomy, has failed. The Rendez Vous technique is another viable option. This method aids endoscopic biliary cannulation by threading a wire anterogradely through the papilla via a surgical, percutaneous, or endoscopic ultrasound (EUS)-guided approach, particularly beneficial when traditional cannulation techniques are unsuccessful or when anatomical alterations occur. As medical science continues to evolve in its understanding and management of biliary adverse events, ERCP-related tools and techniques increasingly become more effective in addressing these issues. Therefore, the primary aim of this observational study is to provide an updated report on the endoscopic management of post-surgical biliary leaks, endeavoring to identify factors related to its efficacy.

## Methods

### Patient cohort

We conducted a double-center retrospective study examining data from the Mediterranean Institute for Transplantation and Highly Specialized Therapies (“ISMETT”) and the Azienda Ospedale-Università Padova (“AOPD”), both of which are internationally recognized as tertiary centers for hepatobiliary disease. A comprehensive electronic search was performed to identify patients who had undergone at least one ERCP, from January 2018 to August 2023 in ISMETT or AOPD. This study was developed following the “Strengthening the Reporting of Observational Studies in Epidemiology (STROBE)” recommendations [12] (Table S3 – Supplementary Materials). All selected cases were subsequently evaluated for inclusion criteria, which was the presence of at least one biliary leak following surgical operation that undergoes at least one ERCP attempt to treat the leak. Exclusion criteria were (1) age inferior to 16 years old; (2) trauma-associated liver leaks; (3) prior biliary procedures unrelated to the current issue between surgery and the leak resolution attempt; (4) over 4 non-endoscopic interventions before the endoscopic attempt; (5) more than 2 missing data per parameter analyzed; and (6) post-procedural follow-up duration shorter than 20 days. Some of the patients came from a District General Hospital (DGH) and were then referred to the two tertiary centers to manage the leak. The primary outcome was the overall endoscopic clinical success, while secondary outcomes were hospital stay length (greater than 5 days) and endoscopy clinical success with the first endoscopic procedure at the tertiary center. Clinical Success was obtained when a treatment proved to be effective in resolving biliary leaks from both a clinical and, if applicable, radiological perspective (no more evidence of leak) (Table S1. “Definitions” – Supplementary Materials), with a timeframe of two weeks. Both outpatients and inpatients were subjected to a follow-up period of at least 20 days.

### Biliary leak

**Fig. 1:**
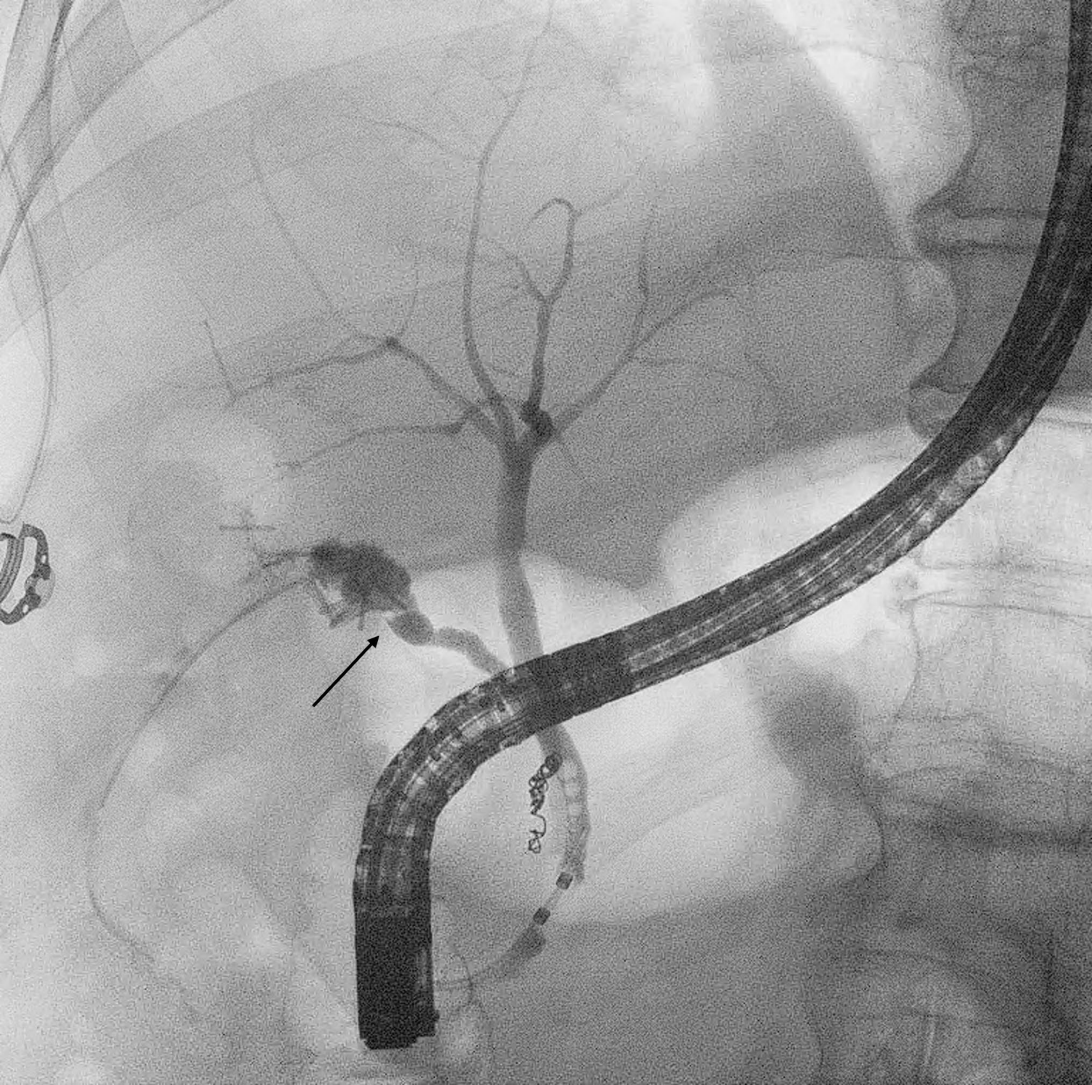
Cholangiography showing a post-cholecystectomy biliary leak from the cystic stump (black arrow)

A biliary leak refers to the escape of bile from any location within the biliary tree (Fig. 1). It is diagnosed by the presence of bile after three days from surgery within the surgical or percutaneous drainage or by radiological evidence of escape of bile or dye from one or more sites of the biliary tree. Resolution of the leak was diagnosed when either abdominal drainage was not actively draining bile, or biliary leak was not seen at radiological examinations (CT-scan or MRI).

Data on the presence of biliary strictures associated with biliary leaks have been collected to evaluate if this association may affect the outcome.

### Interventions

The ERCP (or percutaneous Rendez Vous) was not always the initial treatment employed to address the leak; in some instances, a surgical or percutaneous radiological attempt at correction was undertaken first, based on the assessment of the clinician overseeing the patient. The choice of the percutaneous radiological approach was determined by the radiologist. The decision on the surgical approach was made by the surgeon.

### ERCP technique

Both centers adhered to the latest ESGE recommendations regarding cannulation techniques and the prevention of potential adverse events. For all ERCPs identifying a naïve papilla, a sphincterotomy was carried out to decrease pressure within the biliary tree, facilitating the reversal of bile flow from the leak site to the duodenum. If initial cannulation attempts were unsuccessful, a needle knife sphincterotomy ("Pre-Cut") was performed to gain access to the biliary tree. All metal stents used were fully covered. The polyethylene stents employed had either a 7Fr or 10Fr diameter. Any combined endoscopic-interventional radiological procedure ("Percutaneous Rendez Vous") was considered within the study as an endoscopic approach since in all such cases a biliary prosthesis was inserted endoscopically.

### Statistical analysis

Cohort characteristics, stratified by both endoscopic clinical success (Main Outcome), hospital stay length (greater than 5 days or not—Secondary Outcome), and endoscopy clinical success with the first endoscopic procedure at the tertiary center (Secondary Outcome) were presented as means with standard deviations (SD) for continuous variables and as frequencies with percentages for categorical variables. Missing data were considered as "not applicable" during analysis. The chi-square test was employed for categorical variables in univariate analyses, while a binary logistic regression model was used to examine the relationship between the continuous variables and the outcomes. For the Primary Outcome, multivariate analyses utilized a logistic regression model, drawing from significant findings in the univariate analysis and adjusting for potential confounders. All statistical computations were executed using the "R" software. A *p* value of < 0.05 was considered statistically significant.

## Results

### Patient characteristics (Table 1)

**Table 1:** Patients’ characteristics

	*n* (%) or mean (SD)
Sex	
Male	41 (63%)
Female	24 (37%)
Age (years)	58 (± 15.3)
Age	
< 60 years old	28 (44%)
≥ 60 years old	36 (56%)
ASA	
< 3	18 (28%)
≥ 3	47 (72%)
Surgical procedure	
Cholecystectomy	29 (45%)
Liver transplant	21 (32%)
Hepatectomy	9 (14%)
Cholecystectomy plus gallbladder bed resection	6 (9%)
Surgical procedure in DGH	
No	47 (72%)
Yes	18 (28%)
Biliary damage type	
Leak	47 (72%)
Leak + stenosis	18 (28%)
Leak location	
Main biliary duct	15 (23%)
Resection plane or biliary stump (including Cystic Stump and Luschka Duct)	24 (38%)
RHD, LHD or IHBD	6 (9%)
Biliary end-to-end anastomosis	19 (30%)

*n* number, *DGH* District General Hospital, *SD* standard deviation, *RHD* right hepatic duct, *LHD* left hepatic duct, *IHBD* intrahepatic biliary duct

A total of 65 patients were identified (41 males and 24 females; mean age 58 years, ± 15.3; 18 with less than 3 in ASA classification and 47 with more or equal to 3) according to inclusion and exclusion criteria.

### Surgical characteristics (Table 1)

Overall, 29 (45%) underwent laparoscopic cholecystectomy, 21 (32%) underwent liver transplantation, 9 (14%) underwent hepatectomy (minor or major), and 6 (9%) were subjected to cholecystectomy and resection of the gallbladder bed. 18 (28%) of the surgical interventions took place at DGH: 15 patients underwent laparoscopic cholecystectomy and 3 underwent cholecystectomy with gallbladder bed resection (or “extended cholecystectomy”), whereas 47 (72%) of the surgical procedures were performed at the tertiary centers.

### Leak characteristics (Table 1)

In 47 (72%) instances, the surgical procedure resulted in single or multiple biliary leaks. In contrast, in 18 (28%) cases, a biliary stricture accompanied the leaks. The most common leak location was at the resection plane or biliary stump (24 patients, 38%), encompassing the cystic duct stump and Luschka’s duct, followed by end-to-end anastomoses (19 patients, 30%), main biliary duct (15 patients, 23%), and right hepatic duct, left hepatic duct, or intrahepatic bile ducts (6 patients, 9%).

### Treatment characteristics (Tables 2, 3)

**Table 2:** Treatments’ characteristics

	*n* (%) or mean (SD)
Previous DGH treatment attempt	
No	52 (80%)
Yes	13 (20%)
First treatment type at the tertiary center	
Endoscopic approach (or combined)	63 (96%)
Radiological approach	1 (2%)
Surgical approach	1 (2%)
Stent positioning during first endoscopic procedure at the tertiary center	
No	7 (12%)
Yes	51 (88%)
Endoscopic stent type	
Polyethylene	41 (80%)
FC-SEMS	10 (20%)
Endoscopic polyethylene diameter ≥ 10Fr	
No	12 (31%)
Yes	27 (69%)
Endoscopic stent location	
Not leak-bridging	15 (31%)
Leak-bridging	34 (69%)

*n* number, *SD* standard deviation, *DGH* District General Hospital, *FC-SEMS* fully covered-self-expandable metallic stent

**Table 3:** Treatments’ outcome

	*n* (%) or mean (SD)
Technical success with first endoscopic treatment at the tertiary center	
No	8 (12%)
Yes	57 (88%)
Clinical success with first endoscopic treatment at the tertiary center	
No	21 (32%)
Yes	44 (68%)
Adverse event with first endoscopic treatment at the tertiary center	
No	59 (91%)
Yes	6 (9%)
Number of treatment attempts at the tertiary center	1.65 (± 1.15)
Overall endoscopic clinical success	
No	8 (12%)
Yes	57 (88%)
Timing surgery-endoscopic treatment at the tertiary center *(days)*	46 (± 100)
Timing surgery-clinical success *(days)*	59.81 (± 112)
Hospital stay > 5 days	
No	17 (26%)
Yes	48 (74%)

*n* number, *SD* standard deviation

In 63 patients (96%), once managed by the tertiary center, endoscopic or combined treatment was performed as the initial approach (61 cases of endoscopic approach and 2 cases of percutaneous Rendez Vous); in the other two cases, percutaneous radiological and surgical treatments were respectively indicated. 13 (20%) of the patients had undergone at least one attempt (endoscopic or surgical) to correct the leak at the DGH before being managed at the tertiary center. In 5 (8%) of the patients, at least one percutaneous Rendez Vous was performed; in all of these cases, an endoscopic stent was placed.

Regarding the first endoscopic procedure performed at the tertiary center, technical success was achieved in 57 patients (88%), and clinical success was also achieved in 44 (68%). Post-procedural adverse events (AEs) during the first endoscopic procedure at the tertiary center occurred in 6 cases (9%): 2 cholangitis, 3 mild pancreatitis, and 1 minor bleeding requiring red blood cell transfusion. All these events have been managed conservatively. Considering the population in which technical endoscopic success was achieved during the first procedure at the tertiary center, in 51 patients (88%), a biliary stent was placed, with 41 (80%) being polyethylene and 10 (20%) being fully covered metallic stents. Among the polyethylene stents, the diameter measurements were 7Fr in 12 (31%) patients and 10Fr in 27 (69%) patients. The stents were positioned "leak-bridging" in 34 patients (69%).

Considering all performed procedures, endoscopic clinical success was achieved in 57 (88%) patients. The average number of endoscopic, radiological, or surgical treatments per patient was 1.65 (± 1.15). The period between the surgical procedure and the first endoscopic treatment at the tertiary center was 46 (± 100) days. The period between the surgical procedure and clinical success was 59.81 (± 112) days. Lastly, 48 patients (74%) had a hospital stay longer than 5 days.

### Primary outcome (Tables 4, 5)

**Table 4:** Univariate analyses (primary outcome)

	Overall endoscopic clinical fail – *n* (%) or mean (SD)	Overall endoscopic clinical success—*n* (%) or mean (SD)	*p* value
Sex			0.669
Male	4 (10%)	37 (90%)	
Female	4 (17%)	20 (83%)	
Age			0.446
< 60 years old	2 (7%)	26 (93%)	
≥ 60 years old	6 (17%)	30 (83%)	
ASA			1
< 3	2 (11%)	16 (89%)	
≥ 3	6 (13%)	41 (87%)	
Surgical procedure			0.553
Cholecystectomy	4 (14%)	25 (86%)	
Liver transplant	1 (5%)	20 (95%)	
Hepatectomy	2 (22%)	7 (78%)	
Cholecystectomy and gallbladder bed resection	1 (17%)	5 (83%)	
Biliary damage type			**0.005**
Leak	2 (4%)	45 (96%)	
Leak + stenosis	6 (33%)	12 (67%)	
Leak location			**0.038**
Main biliary duct	5 (33%)	10 (67%)	
Resection plane or biliary stump (including Cystic Stump and Luschka Duct)	1 (4%)	23 (96%)	
RHD, LHD or IHBD	0	6 (100%)	
Biliary end-to-end anastomosis	2 (10%)	17 (90%)	
Technical success with first endoscopic treatment at the tertiary center			**<0.001**
No	6 (75%)	2 (25%)	
Yes	2 (4%)	55 (96%)	
Timing between surgery and first endoscopic procedure at the tertiary center	95.5 (± 205)	39.5 (± 75)	0.809

Bold values were used to enhance values of *p*-value lesser than 0.05

n, number; SD, standard deviation; RHD, right hepatic duct; LHD, left hepatic duct; IHBD, intrahepatic biliary duct

**Table 5:** Multivariate analyses

	OR (95% CI)	*p* value
Biliary damage type		
Leak	–	–
Leak + stenosis	**0.053** (0.001–0.670)	**0.046**
Technical success with first endoscopic treatment at the tertiary center		
Yes	–	–
No	**0.007** (0.001–0.101)	**0.002**
Sex		
Female	–	–
Male	8.278 (0.557–396.8)	0.176
Age		
< 60	–	–
≥ 60	0.234 (0.007–3.383)	0.322

Bold values were used to enhance values of *p*-value lesser than 0.05

*OR* odds ratio, *CI* confidence interval

Within the study population, males exhibited a higher likelihood of endoscopic clinical success (90% vs 83%, *p* value 0.669) as did patients younger than 60 years old (93% vs 83%, *p* value 0.446). Regarding surgical intervention, patients who underwent liver transplantation showed a higher likelihood of success (95%) compared to those who had a laparoscopic cholecystectomy (86%), cholecystectomy with resection of the gallbladder liver bed (83%), or generic hepatectomy (78%), although this result did not achieve statistical significance. Patients with one or more biliary leaks had a higher likelihood of endoscopic clinical success than those with associated strictures (99% vs 67%, *p* value 0.005). The leak site with the best response to endoscopic treatment was represented by the “right hepatic duct” (RHD), “left hepatic duct” (LHD), or “intrahepatic biliary duct” (IHBD) group (100%), followed by the resection plane or biliary stump, including cystic stump and Luschka duct group (96%), and the end-to-end anastomosis group (90%). Patients with the least likelihood of leak resolution via endoscopy were those with leaks at the main biliary duct level (67%). This correlation achieved statistical significance (*p* value 0.038).

Achieving technical success in the first endoscopic treatment increased the likelihood of clinical success, considering even subsequent procedures (96% vs 25%, *p* value < 0.001). The group with endoscopic clinical success had a lower mean period (in days) between the surgical procedure and the first endoscopic treatment at the reference center compared to the group with clinical failure (39.5 vs 95.5, *p* value 0.140). The subsequent multivariate analysis confirmed that the presence of associated leak and strictures and the lack of technical success during the first endoscopic procedure at the tertiary center were less likely to achieve endoscopic clinical success (aOR 0.053 with *p* value 0.046 and aOR 0.007 with *p* value 0.002, respectively), even after adjusting for gender and age.

### Secondary outcome (Table S2—Supplementary Materials—and Table 6)

**Table 6:** Univariate analyses (secondary outcome – endoscopic clinical success with first endoscopic procedure at the tertiary center)

	Endoscopic clinical fail – *n* (%)	Endoscopic clinical success—*n* (%)	*p* value
Sex			0.889
Male	14 (34%)	27 (66%)	
Female	7 (29%)	17 (71%)	
Age			0.172
< 60 years old	6 (21%)	22 (79%)	
≥ 60 years old	15 (40%)	22 (60%)	
Endoscopic stent type			0.428
Polyethylene	10 (24%)	31 (76%)	
FC-SEMS	1 (10%)	9 (90%)	
Endoscopic polyethylene stent diameter = 10Fr			0.708
No	4 (33%)	8 (67%)	
Yes	7 (26%)	20 (74%)	
Endoscopic stent location			**0.005**
Not leak-bridging	7 (47%)	8 (53%)	
Leak-bridging	3 (9%)	31 (91%)	

Bold values were used to enhance values of *p*-value lesser than 0.05

*n* number, *SD* standard deviation, *FC-SEMS* fully covered-self-expandable metallic stent

Within the study population, males exhibited a higher likelihood of having a hospital stay length > 5 days (88% vs 67%, *p* value 0.474) such as patients with ASA ≥ 3 (79% vs 61%, *p* value 0.258). Patients with a biliary leak following a cholecystectomy combined with resection of the gallbladder bed all had a hospital stay > 5 days (100%). This was in contrast to patients following liver transplantation (76%), laparoscopic cholecystectomy (69%), or other hepatectomy (67%). However, these findings did not achieve statistical significance (*p* value 0.426). In the end, patients who did not receive an endoscopic stent insertion exhibited a higher likelihood of hospital stay length > 5 days (87.5% vs 67%, *p* value 0.465).

Regarding the first endoscopic procedure at the tertiary center, the FC-SEMS showed a higher but not statistically significant endoscopic success than the polyethylene stent (90% vs 76%, *p* value 0.428). The stent placement site was statistically significant: procedures, where the stent was placed leak-bridging, exhibited higher endoscopic success rates (91% vs 53%, *p* value 0.005).

## Discussion

Liver and biliary tract surgery encompasses an extensive area of surgical practice. A common concern across this broad spectrum is the risk of injury to the biliary tract. This can manifest as leaks, strictures, or a combination of both, affecting one or multiple points along the biliary tract. The location and extent of the injury have significant implications for the patient’s prognosis. Most cases require an interventionist medical approach, be it endoscopic, radiological, or surgical. In the presence of leaks, with or without associated strictures, the effectiveness of endoscopic treatment hinges on reducing the resistances in the biliary tree to favor the flow of bile through the duodenum instead of through the leak site. This is achieved through sphincterotomy and/or the placement of a biliary stent. Indeed, our study retrospectively analyzed the cases managed at two referral centers. The results confirm the excellent capabilities of endoscopic (or combined) treatment in resolving such defects: endoscopic clinical success was achieved in 88% of patients; in 69% of patients, success was achieved on the first endoscopic attempt. The remaining group required one or more additional attempts, sometimes in a combined form (Rendez Vous, which has never been chosen as the initial approach). This finding aligns with existing literature data: Adler et al. [13], analyzing a large cohort of 518 patients with PSBL treated via ERCP, reported an endoscopic clinical success rate of 93.4%, with 89.2% achieving success after the first ERCP. The observed difference might also be attributed to patient selection: 20% of the patients in our study were referred by DGH and had already undergone at least one unsuccessful endoscopic treatment attempt. Moreover, in our study, 9% of the patients experienced post-procedural AEs after the first endoscopic procedure at the tertiary center, none of which led to mortality.

Sometimes, endoscopic procedures achieve immediate and complete resolution of a fistula, while other times they result in a progressive reduction monitored by the clinician through a peri-leak "spy" drain or clinical observation. In this regard, the authors believe two aspects warrant further investigation. Firstly, the role of peri-leak abdominal drainage: being an external drainage, it could potentially keep the fistula open due to the lower pressure in the collection sac, despite its monitoring function. The authors routinely remove the drainage within 48 h post-endoscopy, when it is considered not active, meaning that the drainage has highly reduced, even if the leak is not entirely resolved. Secondly, the point at which the endoscopic approach may be considered futile needs attention: Tewani et al. [14] say that post-cholecystectomy leaks benefit from repeat ERCP, while there is no advantage to further endoscopic therapy in persistent leaks after hepatobiliary surgery, so early alternative interventions should be considered. However, these data unfortunately do not provide a detailed analysis of the type of surgery (hepatectomy, OLTx, construction of biliary anastomosis, etc.). In our study, 4–5 endoscopic/combined attempts were sometimes required before achieving clinical endoscopic success, despite the surgical procedure; however, in cases of multiple unsuccessful endoscopic attempts, patients underwent surgical intervention for leak resolution. The author’s approach, based on experience, is to perform at least one endoscopic attempt and, if unsuccessful, a subsequent Rendez Vous attempt. If clinical success is still not achieved, a multidisciplinary discussion is recommended to assess the benefits of a potential surgical approach, which by definition is invasive and often extremely complex. Our study highlighted a clear element, explained by the "Venturi Effect" [15]: biliary leaks associated with biliary stricture have a lower probability of clinical endoscopic success (67% vs. 96%; *p* value 0.005). This finding was further reinforced using a multiple logistic regression model. Strictures may be due to surgical errors (e.g., intraoperative clip placement or inadequate anastomosis) or inflammatory outcomes. We also observed that leaks in the main biliary duct (common bile and hepatic duct) had a reduced endoscopic correction rate (67%) compared to leaks in other parts of the biliary tree, such as resection planes or biliary stumps (including Cystic Stump and Luschka Duct) (96%), Right Hepatic Duct, Left Hepatic Duct, or other Intrahepatic Biliary Ducts (100%), and end-to-end anastomosis sites (90%) (*p* value 0.038). This may be partly explained by the higher flow rate in the main biliary duct. According to Tewani et al. [14], the location of the bile leak (BL) is the most significant factor influencing the success of ERCP therapy, whereas the analysis is conducted between a group with PSBL originating from the cystic duct or the duct of Luschka versus BLs originating from all other sites. Our study not only confirms this finding but also attempts to go a step further by comparing all possible leak sites.

The study found no statistically significant relation between endoscopic clinical success with the first attempt at the tertiary center and either the placement of a biliary stent, its diameter, or material, even if FC-SEMS timidly and not significantly increased clinical success compared to polyethylene stents, independently from diameter (7 or 10 Fr), but further studies with larger cohort are needed to properly evaluate this last association. Moreover, our study suggests that the placement of a leak-bridging stent, when feasible, increases the likelihood of clinical success (91% vs. 53%, *p* value 0.005). In this context, Vlaemynck et al. [16] recommended sphincterotomy with stenting if the biliary leak can be bridged. However, our study did not show a statistically significant relation between clinical endoscopic success and the timing between surgery and the first endoscopic procedure at the tertiary center. This parameter was, on average, longer in patients who did not achieve clinical endoscopic success. A reduced timing might imply a lesser presence or consolidation of inflammatory processes affecting the bile duct wall at the leak site or the development of an associated stricture. The bicentric and retrospective design of this study inherently brings forth several limitations necessitating thoughtful consideration. Firstly, the absence of standardized diagnostic and therapeutic protocols in the two participating centers may compromise the consistency and reproducibility of the methods employed. However, it is crucial to highlight that, during the timeframe of the study, both institutions were in strict compliance with the most recent European guidelines available [17–19]. Another fundamental constraint arises from the study’s framework that precludes direct comparisons between the outcomes of endoscopic corrections and those obtained through surgical or radiological interventions. Similarly, we are limited in our capacity to contrast the results of endoscopic procedures carried out at tertiary centers with those conducted at DGH. Another limitation is the relatively modest sample size we engaged within this study, which is an obvious consequence of the restrictive criteria that were applied in the context of a retrospective design. This restrictive cohort dimension possibly underlies the observed lack of statistical significance in some correlations, notably those concerning the interval between surgery and the first endoscopic interventions at the tertiary center. Such a limited dataset may also circumscribe our ability to fully exploit multivariate analyses for more in-depth insights. Contemplating these limitations, the value of launching subsequent research attempts, ideally with a prospective layout and encompassing a more expansive and uniform patient cohort, becomes indisputable. Furthermore, the decision to incorporate data from patients who underwent liver transplantation was made in line with our overarching objective: to identify common factors influencing the likelihood of success in all post-surgical biliary leaks. There is a notable absence of robust studies in this area in the literature (25); hence, this concept might benefit from further validation through more focused trials.

## Conclusion

In conclusion, our study confirms endoscopic treatment is safe and effective for treating PSBL, regardless of the type of preceding surgery. The location of the post-surgical bile leak impacts the likelihood of clinical success, as does the presence of an associated stricture in case of endoscopic clinical success. Finally, biliary stenting is fundamental to resolving leaks, and, if possible, the stent should be placed leak-bridging to enhance treatment efficacy. Our findings are only partially reported in the existing literature; hence they represent a novel contribution.

## Supplementary Information

Below is the link to the electronic supplementary material.

Supplementary file1 (DOCX 34 KB)
